# Accreditation of medical education in Brazil: an evaluation of seventy-six medical schools

**DOI:** 10.1186/s12909-024-05623-8

**Published:** 2024-06-12

**Authors:** Patricia Tempski, Leticia C. Girotto, Sigisfredo Brenelli, Donizeti D. Giamberardino, Milton A. Martins

**Affiliations:** 1 Sistema de Acreditação de Escolas Médicas, Conselho Federal de Medicina, Brasilia, Brazil; 2 Centro de Desenvolvimento de Educação Médica da Faculdade de Medicina, da Universidade de São Paulo, Av. Dr. Arnaldo 455 sala 1210, Sao Paulo, 01246-903 Brazil; 3 Hospital Pequeno Príncipe, Curitiba, Brazil

**Keywords:** Accreditation of medical schools, Social accountability, Continuous quality improvement, Teaching in health system

## Abstract

**Background:**

We present the first results of the Accreditation System of Medical Schools (Sistema de Acreditação de Escolas Médicas – SAEME) in Brazil.

**Methods:**

We evaluated the results of the accreditation of medical schools from 2015 to 2023. The self-evaluation form of the SAEME is specific for medical education programs and has eighty domains, which results in final decisions that are sufficient or insufficient for each domain. We evaluated the results of the first seventy-six medical schools evaluated by the SAEME.

**Results:**

Fifty-five medical schools (72.4%) were accredited, and 21 (27.6%) were not. Seventy-two (94.7%) medical schools were considered sufficient in social accountability, 93.4% in integration with the family health program, 75.0% in faculty development programs and 78.9% in environmental sustainability. There was an emphasis on SAEME in student well-being, with seventeen domains in this area, and 71.7% of these domains were sufficient. The areas with the lowest levels of sufficiency were interprofessional education, mentoring programs, student assessment and weekly distribution of educational activities.

**Conclusion:**

Medical schools in Brazil are strongly committed to social accountability, integration with the national health system, environmental sustainability and student well-being programs. SAEME is moving from episodic evaluations of medical schools to continuous quality improvement policies.

**Supplementary Information:**

The online version contains supplementary material available at 10.1186/s12909-024-05623-8.

## Background

There has been a substantial increase in the accreditation of medical schools in many countries in recent years [8]. It has been suggested that accreditation of medical education results in competent practicing doctors [10, 12, 29, 30]. However, there are few data showing evidence that the accreditation of medical schools has a positive impact on the quality of medical education[15, 31, 33].

The World Health Organization recommends that countries establish accreditation bodies for quality assurance in medical education. The World Federation for Medical Education (WFME) has a program for the recognition of accreditation bodies for basic medical education in each country as one of its most important projects [20, 32].

The System of Accreditation of Medical Schools (Sistema de Acreditação de Escolas Médicas – SAEME) was established in 2015 in Brazil and offers voluntary accreditation of undergraduate medical education programs [1]. In 2019, SAEME became the first accreditation system in South America recognized by the World Federation for Medical Education (WFME).

SAEME rapidly became one of the largest accreditation agencies in the world, since, until 2023, seventy-six medical schools were first evaluated. Brazil has very diverse regions with 389 medical schools within Brazil alone. Additionally, there are enormous challenges for SAEME to apply high-quality standards, respect the different missions of medical schools and help medical schools to be socially accountable and contribute to the development of the Brazilian National Health System.

We describe some of the main characteristics of SAEME, their impact on medical education in Brazil and some present and future challenges in contributing to the quality and social accountability of medical schools.

## Methods

SAEME was established by an agreement between the Brazilian Federal Medical Council (Conselho Federal de Medicina—CFM), the Brazilian public agency responsible for professional regulation and medical licensing, and the Brazilian Association of Medical Education (Associação Brasileira de Educação Médica – ABEM) and, in 2022, became a Department of CFM.

The process of accreditation by SAEME is voluntary, and medical schools are eligible for accreditation if they are officially recognized by the Ministry of Education of Brazil or other state regulatory agencies, are not involved in any legal proceedings, and have graduated from at least one class.

To construct the accreditation standards of SAEME and the self-report form, Tempski et al. reviewed standards of various international organizations: the WFME, Liaison Committee on Medical Education of the USA and Canada, the Australian Medical Council, the General Medical Council of the United Kingdom, the Nederlands-Vlaamse Accreditation Organization, the Japonese National Institution for Academic Degrees and University Evaluation and the Standards for Accreditation of Mercosur. They also studied the Brazilian Guidelines for Medical Education and the Sistema Nacional de Avaliação do Ensino Superior (National System for Evaluation of Higher Education) of the Ministry of Education to ensure that the standards were consistent with relevant government policies [34].

Tempski et al. performed a pilot study evaluating eight medical schools. The standards were then adapted and submitted for professional and public review. The final standards were adopted in 2015 [35].

The SAEME requires that medical programs follow the National Guidelines for Medical Education of Brazil. These guidelines, developed by the Ministry of Education, ensure that medical school graduates are prepared to meet the needs of the Brazilian National Health System. SAEME has five core values: high standards of quality, independence, transparency, ethics and social accountability.

The standards for the accreditation of SAEME are specific to medical education programs and cover five areas (educational management, educational programs, academic staff/faculty, students, and educational resources). The domains (*n* = 80), translated into English, are supplied in Appendix A and are also available on the SAEME website (www.saeme.org.br). SAEME has developed a database tool where assessors can input information concerning each of the 80 standards/domains. The types of evidence required to meet each standard are also supplied. All domains require a self-evaluation of the medical school and the provision of evidence [1].

The survey team comprises four people: three faculty members and one medical student. One of the faculty members—the evaluation coordinator—is the team leader. Two members must be medical doctors, and the third must be a health professional involved in medical education. The role of team leader is important in the scheduling and preparation for the visit, leading the survey team during the visit and leading in the drafting of the report.

Medical school visits include meetings with the dean, curriculum committee, research committee, students (a focus group with randomly selected students and a plenary session with students), academic staff (a focus group with randomly selected faculty and a plenary session with faculty), student support services, and representatives of local health authorities. The survey team directly observes the facilities and resources at the school and clinical training sites.

All evaluators of SAEME participate in training and continuing education processes. There is an initial two-day training for new assessors, which includes the discussion of real cases. There is also an annual retraining requirement, with two meetings scheduled per year, with all members of survey teams required to attend one of them.

SAEME requires that an accredited medical school submit a report every three years describing any major changes or significant modifications to the educational program (e.g., curriculum reform). SAEME requires prior notification of changes that affect the medical education program. SAEME determines, based on the documented changes (or intent to change), whether further evaluation is needed. SAEME can decide to order new information and/or documents from medical school or to perform a new (focused) site visit. At the end of an accreditation period (six years), the school must restart the process with a new application for accreditation.

The final decision of the accreditation process is provided by an accreditation committee composed of experienced faculty from diverse medical schools in Brazil. The final decision is based on the number of domains that were considered sufficient or insufficient, in the self-reports of medical schools, the reports of the visiting team and the reports of the General Secretariat of SAEME.

We reviewed the decisions of the Accreditation Committee of SAEME concerning domains, whether they were sufficient or insufficient, and evaluated the strong aspects of SAEME and the main challenges observed in medical schools in Brazil.

## Results

### Accredited medical schools

From 2015 to 2023, seventy-six medical schools were evaluated for the first time; 55 (72.4%) were accredited, and 21 (27.6%) were not. Medical schools that were not accredited could start a new process of accreditation within one year. During the worst years of the COVID-19 pandemic (2020 and 2021), SAEME evaluated only eleven medical schools. The visiting team visited the medical school in person, but the meetings with faculty and students were remote.

Table 1 shows the number of medical school eligible for accreditation and the percentage of schools that were evaluated and accredited in the five geographic regions of Brazil. We also show the area and the population of these five regions. The north region is the largest one, with lower population and less medical schools. In contrast, the southeast region has the higher population and more medical schools. From 2015 to 2023, 25.9% of eligible medical schools (at least seven years of existence) were evaluated and this percentage varied from 15.2% in the Northeast region to 34.1% in the South region of Brazil.

**Table 1:** Medical schools evaluated by SAEME in the five geographic regions of Brazil

	% total area	% total population	Medical schools	Evaluated by SAEME	Accredited
North	45.3%	8.5%	26	5 (19.2%)	4 (80.0%)
Northeast	18.1%	26.9%	79	12 (15.2%)	9 (75.0%)
Central-western	18.9%	8.0%	25	6 (24.0%)	4 (66.7%)
Southeast	10.9%	41.8%	120	38 (31.7%)	27 (71,1%)
South	6.8%	14.7%	44	15 (34.1%)	11 (73.3%)
Total			294	76 (25.9%)	55 (72.4%)

Total area of Brazil: 8,515,758 Km^2^; total population: 203,062,512 (2022 census)

Table 2 shows the number of private and public medical schools that were evaluated and the percentage that was accredited.

**Table 2:** Public and private medical schools evaluated and accredited by SAEME

	Number of medical schools evaluated	Percentage of medical schools that were accredited
Public medical schools	30	73.3%
Private medical schools	46	67.5%
Total	76	72.4%

### Social accountability

Domain 1.1 of social accountability is sufficient when: “The academic institution promotes actions that contribute to the improvement of living conditions of local and regional communities, especially in the areas of education and health.” The medical school must “Describe actions that express the social accountability of medical school and how these actions are recognized by society.” Seventy-two (94.7%) medical schools were considered sufficient for this domain.

There is another domain in self-study that requires that medical schools show good integration with the health system of Brazil, especially with the basic health units where family health teams work (Domain 5.11). This domain is considered sufficient if “there are health care units and family health centers in sufficient number, integrated to the health system, with adequate infrastructure for teaching and sufficient family health teams and preceptors considering the learning objectives of the curriculum. These units provide an appropriate environment for medical education and training.” Considering this domain, seventy-one medical schools (93,4%) were sufficient.

### Faculty development policies

Domain 1.12 (institutional actions designed to promote faculty development) is sufficient when “There is an institutional process in place to promote the development of academic competences, including teacher training, development, support, appraisal, and participation in medical education events and courses, in addition to the actions implemented by the faculty development committee.” Considering this domain, 57 medical schools were sufficient (75.0%), and 19 were not (25.0%).

### Standards related to medical students (Standards 4)

SAEME places a strong emphasis on student well-being, with a total of seventeen domains that, in general, establish the following: “Medical school promotes a healthy educational environment, positive for learning and personal development. Medical schools promote a culture of institutional resilience, with values of gratitude, generosity, respect, and honesty. It provides students with conditions of permanence, health promotion and prevention, access to health services, and psychological and pedagogical support. Medical schools have clear policies for admission, transfer, and student mobility. Students have representation and participate in governance, design, and evaluation of educational programs. Medical school approves of the presence of representative student organizations and provides proper physical space for them.” Table 3 shows the results (sufficient/insufficient) of all these domains.

**Table 3:** Results of the evaluation of seventy-six medical schools by the System of Accreditation of Medical Schools: domains of medical students

Domain	Number of medical schools sufficient	Number of medical schools insufficient
4.5 Student transference	74 (97.4%)	2 (2.6%)
4.8 Right of student inquiry	72 (94.7%)	4 (5.3%)
4.10 Student organizations	69 (90.8%)	7 (9.2%)
4.1 Selection and admission process	68 (89.5%)	8 (10.5%)
4.9 Representativeness	68 (89.5%)	8 (10.5%)
4.2 Welcome program	64 (84.2%)	12 (15.8%)
4.4 Scholarships	61 (80.3%)	15 (19.7%)
4.6 Student mobility	60 (78.9%)	16 (21.1%)
4.12 Preventive health care	57 (75.0%)	19 (25.0%)
4.15 Psycho-pedagogical support	55 (72.4%)	21 (27.6%)
4.14 Mental health care	55 (72.4%)	21 (27.6%)
4.7 Institutional policies of nondiscrimination	52 (65.6%)	24 (34.4%)
4.11 Participation in meetings and congresses	44 (57.9%)	32 (41.1%)
4.12 Health care	42 (55.3%)	34 (44.7%)
4.3 Programs to support student permanence	33 (45.3%)	43 (54.7%)
4.17 Quality of life programs	33 (43.4%)	43 (56.3%)
4.16 Mentoring programs	20 (26.3%)	56 (73.7%)
Mean ± Standard deviation	54.5 ± 15.4	21.5 ± 15.4

For a detailed description of each domain, see Appendix A

### Environmental sustainability

Sixty medical schools (78,9%) were considered sufficient in this domain (Domain 5.18), providing evidence of institutional policies for environmental sustainability.

### Domains with a greater number of “insufficient”

Table 4 shows the four domains that showed higher percentages of insufficient scores when evaluated by the Accreditation Commission of SAEME.

**Table 4:** Domains of SAEMs with a greater number of “insufficient”

Domain	Number of medical schools sufficient	Number of medical schools insufficient
2.6 Interprofessional education	13 (17.1%)	63 (82.9%)
4.16 Mentoring programs	20 (26.3%)	56 (73.7%)
2.17 Weekly schedule of learning activities	27 (35.5%)	49 (64.5%)
2.9 Student assessment	33 (43.4%)	43 (56.6%)

For a detailed description of each domain, see Appendix A

## Discussion

Undergraduate medical education in Brazil is a six-year program. There has been an increase in the number of medical schools in Brazil in recent years, and there are currently 389 medical schools in Brazil. However, to apply for the accreditation of SAEME, medical schools must have at least one year of graduates, and these initial results can provide a good picture only of medical schools that have been operating for more than six years (294 medical schools). Brazil also has a system for the regulation of superior education, established by federal law in 2004 and led by the Ministry of Education (Sistema de Avaliação do Ensino Superior – SINAES), which has the power to authorize the opening and closing of educational institutions and courses and to require universities and other institutions to comply with recommendations.

There is an unequal distribution of medical schools across Brazilian territory (Table 1). The North region of Brazil where the Amazon Forest is situated, has a huge area, low population compared to other regions and less medical schools. SAEME also did not have a homogenous impact on Brazilian medical schools with a higher percentage of medical schools in the Southeast and South regions of Brazil that applied for the accreditation process compared to the other three regions (Table 1). Interestingly, the percentage of medical schools that were accredited was similar in all geographic regions of the country (66.7 to 80.0%), suggesting that, despite the very important regional differences in Brazil, the quality of medical schools that applied for accreditation by SAEME was similar considering the five different geographic regions of Brazil.

In the recent years, the increase in the number of private medical schools was greater than public medical schools. Scheffer al [26] showed that, in 2022, there were 121 public and 268 private medical schools in Brazil. Although more private medical schools applied for accreditation (46, compared to 30 public medical schools), the percentage of public medical schools was greater. Interestingly, the percentage of accreditation of public and private medical schools was similar (Table 2).

We chose to analyze domains considered important to the needs of Brazilian society and also the international literature (social accountability, integration with the health system, faculty development policies and environmental sustainability) [2, 9, 16]. We also presented detailed description of student’s domains, that we consider one of the stronger aspects of SAEME.

One important result of our study is that medical schools in Brazil are committed to social accountability. In fact, 94.7% of the medical schools that were evaluated by the SAEME were considered sufficient for this domain.

According to Boelen and Woollard [9], “Medical schools must demonstrate a consistent commitment to social accountability in their formal programs and in their ‘hidden curricula’. Through effective engagement with collaborative partners, they must focus their education, research, and service resources on the pursuit of understanding and addressing the priority health concerns of their societies.” SAEME uses a similar concept of social accountability (Appendix A). We suggest that the excellence of social accountability should be included in all accreditation systems worldwide [22].

Brazil has a National Health System that covers all its population. In 1988, the Brazilian Constitution defined health as a universal right and a state responsibility, and the Unified Health System (Sistema Único de Saúde—SUS) was created in 1990. With successes and setbacks in the implementation of health programs and the organization of its health system, Brazil has achieved nearly universal access to health-care services for the population [13, 14, 23]. There was a dramatic increase in health service coverage in only three decades. One of the main reasons for the successes of the SUS is the Programa Saúde da Família (PSF), or the Family Health Program, which was implemented a few years after the creation of the SUS. This program now provides comprehensive primary care services in 95% of all municipalities, covering more than 55% of the population—more than 85 million people. PSF is based on multidisciplinary teams comprising a doctor, a nurse, a nurse auxiliary, and four to six community health workers. PSF is a good example of a rapidly scaled-up, comprehensive primary care system [13, 23].

Medical schools must contribute to the development of the SUS and medical students must be included in health care teams. This requirement of medical school is included in Domain 5.11, which requires that medical schools are present in a sufficient number of health care units and family health teams with adequate infrastructure for teaching. Our finding that 93.4% of medical schools evaluated by SAEME were sufficient in this domain provides additional evidence of the commitment of medical schools to the national health system in Brazil.

According to Amaral and Norcini [5], one important difference in standards between the United States and Brazil is the integration of medical education and the public health care system. In the United States, there are no similar requirements, and population health curricula vary according to school size and funding [5]. However, it is important to consider the intrinsic connection between the social accountability of medical schools and the compromise with the local health system.

Since its creation, SAEME considers it very important that medical schools have systems for supporting students and including medical students in the governance of medical schools and in discussions about curricula and teaching methods. We decided to include a medical student on the visit team. Candidate medical students are selected and participate in a training program and are supervised by the coordinator of the team. The visit team and the deans of medical schools provide very positive feedback on the medical students on the team.

Medical schools must promote a healthy educational environment that is positive for learning and personal development [2, 21]. This objective has been partially achieved by medical schools (Table 1). In fact, 54.5 ± 15.4 of the 76 medical schools evaluated in seventeen domains (71.7%) were “sufficient” in the student domain.

Knight et al. [21] called for the expansion of accreditation requirements in medical schools to include medical student well-being. We observed that the percentages of medical schools that were evaluated by SAEME and were considered sufficient in the domains of the functioning of a mental health care system and preventive health care programs were 72.4% and 75.0%, respectively. The visiting teams of SAEME noted the substantial effort of medical schools to establish comprehensive programs for the well-being of medical students and a good educational environment.

We also observed that 65.6% of medical schools have institutional policies of nondiscrimination (Domain 4.7, “Medical school has institutional policies against any discrimination considering gender, sexual orientation, ethnicity, religious beliefs, age, citizenship and socioeconomic status and there are effective programs and actions”). This observation is evidence that medical schools in Brazil are contributing to equity, diversity, and inclusion, as recommended by various international societies of medical education [3, 6, 11, 24, 25]. In addition, many affirmative action programs are being implemented in Brazil, both in public and private higher education institutions.

We observed that 57 medical schools were sufficient (75.0%) considering faculty development programs. Faculty development has become an increasingly important component of medical education, and most medical schools now offer formal faculty development programs and activities.

We observed that most medical schools (78.9%) contribute to environmental sustainability. Human health is increasingly threatened by changes in the environment and climate, including rising temperatures, air and water pollution, disease vector migration, floods, and droughts. The American College of Physicians and the Association for Medical Education in Europe, among many other organizations, have published calls for physicians and physicians-in-training to develop basic knowledge of the science of climate change and an awareness of the associated health risks [16, 27]. The development of programs of environmental sustainability in medical schools may stimulate the reflection of medical students and physicians-in-training about their role as professionals and citizens in the future of our planet.

This first analysis of the results of the SAEME allows us to observe the areas where there are more challenges in medical schools in Brazil to reach excellence in medical education (Table 2). The domains for higher percentages of insufficient participants were interprofessional education, mentoring programs, student assessment and weekly distribution of educational activities. These are important areas where improvements in medical schools in Brazil are needed. We observed that many medical schools in Brazil still allow a cognitive workload without allowing enough free time for students. In addition, many schools still need to improve the assessment of medical students [28]. There is evidence that mentorship programs stimulate the reflection of medical students and contribute to the building of an ethical professional identity [36].

Interprofessional education was the worst domain in the medical schools evaluated by the SAEME. According to the World Health Organization guidelines, interprofessional education occurs when students from two or more professions learn about, from and with each other to enable effective collaboration and improve health outcomes [17, 19]. Including programs of interprofessional education in medical curricula is very important for preparing medical students for future work in the Brazilian health system.

It has been considered difficult to evaluate the impact of the accreditation of medical schools [37–39]. Our study shows the status of medical schools in Brazil, more than an impact of accreditation. However, more important than episodic accreditation, the development of a process of continuous quality improvement of medical schools can positively influence the quality of medical education worldwide [4, 7, 18]. The final SAEME report, which was sent to medical schools, details each of the eighty domains evaluated, providing evidence, literature, and suggestions for medical schools. According to deans of medical schools that were evaluated by SAEME, the report became an instrument to guide the reflection and planning of medical schools. We created an area on our website with the report of examples of excellence observed during the accreditation process and that we consider it important to share with other medical schools (saeme.org.br). We also created a council of the deans of accredited medical schools, with periodic meetings to suggest improvements in SAEME and to share concerns and experiences in medical education. SAEME was the first accrediting agency in South America recognized by WFME. This recognition stimulated other agencies to apply to recognition by WFME.

### Supplementary Information

Supplementary Material 1.

## Data Availability

The datasets generated during and/or analyzed during the current study are available from the corresponding author upon reasonable request.
