# Supplementary material for: Accreditation of medical education in Brazil: an evaluation of seventy-six medical schools
Source: BMC Med Educ. 2024 Jun 12;24:656. doi: 10.1186/s12909-024-05623-8 (PMC11167757; doi:10.1186/s12909-024-05623-8)
Supplement: Supplementary file 1 — Supplementary Material 1. [file 12909_2024_5623_MOESM1_ESM.pdf]

SAEME Self-study

The self-study is part of SAEME accreditation process, a joint initiative of the Federal Council of Medicine and the Brazilian Association of Medical Education. It intends to evaluate Medical Schools, their context and institutional policies, curriculum, faculty, students' affairs, and the educational environment.

The objectives of accreditation are to recognize and validate good practices, and to contribute to the improvement of medical education, directing an institutional self-reflection towards quality. This evaluation process applies the concept of sufficiency and insufficiency and it is not classificatory in any way. It also identifies areas or aspects that have achieved academic excellence and areas that require improvement.

This document is divided into three sections: the first one introduces the education institution and the medical school; the second describes the documentation required; the third analyzes the quality of program in five standards and provides evidences.

General information about the Medical School is found under Section I of the Self-study. These data are obtained from the Ministry of Education database (Ministério da Educação e-MEC) and includes the information provided by each medical school (average number of enrolled and graduating students who benefit from Student Grant Funds (PROUNI grant or from the Fundo de Financiamento Estudantil – FIES).

Section II describes the documentation required, which includes the curriculum proposed by the schools, modules and contents, and number of hours allocated for each year/semester. In addition, medical schools are required to fill out detailed spreadsheets including academic planning, faculty and preceptors profile.

Section III describes the standards (5) and their elements (80) used as quality indicators for self- study, which are required to demonstrate proven evidence of validity. The elements are organized into five standards: Educational Management, Educational Program, Academic Staff/Faculty, Students and Educational Resources.

Self-assessment should be a participatory process involving all those involved in management, teaching, assistance and research of the medical course, in addition to the administrative, teaching management and student support sectors.

Section I - MEDICAL SCHOOL INFORMATION

**Sponsoring Institution**	Name:
	Address:
**Educational Institution**	Name:
	Address:
	Company name:
	Legal status: (Public / Private)
	Founded in:
	Undergraduate courses offered in the health area:
	Postgraduate courses offered in the health area:
**Medical School**	Implemented in:
	Number of vacancies available per year:
	Number of students enrolled (average based on the past three years):
	Number of students enrolled benefiting from tuition scholarship, PROUNI, FIES (average based on the past three years):
	Number of graduates (average of last three years):
	Total of hours of program:
	Address:
	Phone number:
	Email:

Section II – MEDICAL SCHOOL DOCUMENTATION

The following documentation must be attached to Section II:

§ Curriculum

§ Curriculum worksheet

§ Academic workload for each year/period of the course

The following worksheets should be completed:

§ Faculty profile

§ Preceptors profile

§ Educational planning

§ Scientific publications

Other pertinent and relevant documents for the course accreditation process may be attached.

Section III – STANDARDS AND EVIDENCES

In the SAEME self-study there are five standards: Educational Management, Educational Program, Academic Staff/Faculty, Students and Educational Resources. For each standard there are elements (total of eighty). For each element, point out the concept (insufficient or sufficient) that better characterizes your program, justify and attach evidences supporting your answer.

Standard 1: Educational Management

Medical school must bear an educational program consistent with the institutional view and mission. The educational program of the medical school should be in accordance with the main health demands of society, should be integrated with the local health system, and contribute to regional development. The educational governance must be democratic and competent to ensure the constant improvement of medical training. The incentive to the development of the faculty and academic staff must be one of the targets of educational governance. The medical school must use a systematic data collection process concerning teaching and learning issues and the academic environment and uses these data to improve the medical course. There must be financial sustainability and projects for improving the educational environment.

**1.1 Social accountability**	
Sufficient	The academic institution promotes actions that contribute to the improvement of living conditions of local and regional communities, especially in the areas of education and public health.
Evidence	Describe actions that express the social accountability of medical school and how these actions are recognized by society.

1.2 Linkage between educational program and health system	
Sufficient	The linkage between educational program with local and regional health system is effective and formalized through a written agreement, addressing the three levels of health care.
Evidence	Provide copy of the written agreements(s) between the medical school and the health system and describe the linkage between educational program and health system.

1.3 Support to public policies	
Sufficient	The educational institution supports public policies of education and health and develops actions that can be demonstrated.
Evidence	Describe the public policies of education and health that the medical school participates or supports.

1.4 Regulation assignment of academic authorities	
Sufficient	There are regulatory procedures for the election or designation of academic authorities, including length of service.
Evidence	Attach the institutional regulation chapter that describes the election or designation of academic authorities and the length of their service.

1.5 Dean of medical school	
Sufficient	The dean has proven professional experience in the medical field and medical education, evidenced by attending medical education meetings in the last three years.
Evidence	Submit a dean’s brief curriculum including his/her degrees, academic production and participation in medical education meetings in the last three years.

1.6. Workload of dean of medical school	
Sufficient	The dean works full time and has sufficient time to fulfill his/her duties concerning medical course.
Evidence	Attach the dean’s work contract and describe each one of his/her responsibilities, including the working hours allocated for each one.

1.7 Council of medical course	
Sufficient	There is a council of medical course elected by its peers, with representatives of cycles, modules and/or academic axes of medical program, has a regulatory basis for the renewal of its members and has representatives of medical students in number equivalent or superior of 10% of the members of the council.
Evidence	Describe the composition of the council of medical course, listing each member by name, area of expertise, and role within the medical school, the procedures to select the council of medical school members and the length of their service.

1.8 Council of medical course meetings	
Sufficient	The participation in the council of medical course is regulated and its meetings are held at least quarterly; there is a record of minutes and decisions corresponding to the past three years.
Evidence	Attach the agenda of the meetings of the council of medical course held over the past three years, and the minutes of the last three meetings.

1.9 Curriculum committee	
Sufficient	There is a curriculum committee formed by at least five teachers of the program, with part-time or full-time academic workload, and at least three of them have specialization in health professions and/or medical education.
Evidence	Submit a brief curriculum of each member of the curriculum committee, describing their expertise in health professions education.

1.10 Curriculum committee activities	
Sufficient	The curriculum committee is regulated and its meetings are held at least bimonthly; there is a record of the decisions made regarding the improvement of the medical curriculum over the past twelve months.
Evidence	Attach the agenda of each curriculum committee meeting of the past twelve months and the minutes of the last three meetings.

1.11 Faculty development committee	
Sufficient	There is a faculty development committee of the institution and/or medical school and the committee is composed of faculty and specialists in health sciences education. This committee has actions to improve teaching skills of teachers.
Evidence	Submit the composition of the faculty development committee including a brief curriculum of each one of its members, describing their experience in health professions education. Describe the recent work of the committee, including the identification of the faculty developing identified priorities and the actions developed to improve teaching skills of faculty members.

1.12 Institutional actions designed to promote faculty development	
Sufficient	There is an institutional process in place to promote the development of academic competences, including teacher training, development, support, appraisal, and participation in medical education events and courses, in addition to the actions implemented by the faculty development committee.
Evidence	Describe the institutional actions concerning faculty development in the last three years.

1.13 Institutional self-evaluation	
Sufficient	The educational institution uses a systematic data collection process concerning teaching and learning issues and the academic environment that considers the perceptions of academic authorities, faculty, students and other members of academic community and the institution uses these data to improve the medical course.
Evidence	Describe the institutional self-evaluation and the impact on the improvement of medical course. Include the results of the last self-evaluation performed by the institution.

**1.14 Improvement of the quality of academic environment**	
Sufficient	The institution has a plan of financing the academic activities that assures financial sustainability and improvement the quality of educational environment and the learning process.
Evidence	Describe the policy of control and improvement of the quality of the academic environment.

Standard 2: Educational Program

The educational program of the medical school has clear educational outcomes, coherent with the health needs of the population. It is detailed and ensures, at the end of the course, the acquisition of the expected knowledge, skills and attitudes in Brazilian National Guidelines for Medical Education. Teachers, other members of academic staff and students know the educational program and its principles. There is vertical and horizontal curriculum integration that includes theoretical and practical aspects of medicine, different areas of knowledge, levels of heath care and includes psychological, social, cultural, economic and environmental determinants of health. The educational program has predominantly teaching methods student-centered and include inter-professional education. The clinical teaching activities contemplate different levels of health care and diversified learning settings and the students have supervision of faculty or preceptors. There are elective, complementary and university extension activities in the curriculum. The educational program continuous and systematic student assessment. The assessment is formative and summative and provides continuous feedback. The program evaluation is continuous and used to improve the curriculum. Patient safety is a fundamental principle of the educational program.

**2.1 Professional profile**	
Sufficient	There is a description of the professional profile of the graduate in the curriculum and this professional profile is coherent to that established by the Brazilian National Guidelines for Medical Education. The professional profile of the graduate is known by faculty and medical students.
Evidence	Describe the professional profile of graduate and how it is coherent to the profile established by the Brazilian National Guidelines for Medical Education. Describe how this profile is known by the academic community.

2.2 Learning outcomes	
Sufficient	The leaning outcomes established by the curriculum are coherent with those stablished by the National Curricular Guidelines including those professional values stablished by these guidelines.
Evidence	Describe the main learning outcomes of the medical course and how these outcomes are coherent to those established by the National Curricular Guidelines for Medical Education of the Ministry of Education.

2.3 Health needs	
Sufficient	There is a process to assure that local and regional health needs contribute to the curriculum, and the curricular changes induced by this process can be identified.
Evidence	Describe the process of identification and incorporation of the epidemiological profile of the community in the design of curriculum. Describe the educational activities in the curriculum that demonstrate that the epidemiological profile of the local and regional community is important in teaching, research and services to the community. Describe the curricular changes that were performed based in the local and regional health needs.

2.4 Curriculum content (Syllabus)	
Sufficient	The curriculum content is coherent with the learning objectives stablished by the curriculum of medical course and it is sufficient to accomplish the professional profile of the graduate. The curriculum content includes individual and community aspects of health as well as health promotion, prevention, diagnosis, treatment and rehabilitation.
Evidence	Provide a critical analysis of the curriculum content (syllabus), highlighting how the balance between individual and community aspects of health care, and promotion, prevention, diagnostic, treatment and rehabilitation contents is present in the curriculum.

2.5 Teaching methods	
Sufficient	The teaching methods are coherent with the learning outcomes of curriculum and are predominantly student-centered and based on professional practice and health needs of the society.
Evidence	Describe the main teaching methods used in the curriculum and their distribution throughout the program. Include a file with photos, videos and/or publications.

2.6 Inter-professional education	
Sufficient	The educational program includes strategies that promote collaborative learning among students of different health professions.
Evidence	Describe the inter-professional learning activities present in the curriculum, including duration, year of medical course and health professions included. Provide photos, videos and/or publications.

2.7 Learning settings	
Sufficient	Educational activities are in diversified learning settings (classrooms, laboratories, tertiary and secondary hospitals, outpatient clinics, primary care units and community, distributed throughout the course.
Evidence	Describe and provide a critical analysis of the distribution of learning settings throughout the medical course, including the advantages and disadvantages of each one. Provide photos, videos, and/or publications.

2.8 Clinical teaching	
Sufficient	Clinical teaching activities contemplate different levels of health care in the following areas throughout the course: internal medicine, surgery, pediatrics, obstetrics-gynecology, family and community medicine, public health, mental health and emergency medicine. They are conducted under the supervision of faculty and/or preceptors with institutional appointment, providing contact to health problems of community since the beginning of medical course. The medical program promotes role modelling as a learning method, particularly in clinical practice and research.
Evidence	Provide a critical evaluation of the characteristics and distribution of clinical teaching activities during medical course. Describe how the faculty are familiar with the learning objectives of the course or clerkship and prepared for their roles in teaching and assessment and as role models.

2.9 Student assessment	
Sufficient	Throughout the medical education program there is a system in place for the assessment of student achievement, that employs a variety of measures (including direct observation), and includes acquisition of the knowledge, clinical skills, behaviors and attitudes specified in the medical program objectives. There is a continuous feedback as part of the assessment. Only those students meeting the competencies and profile established by the educational program are awarded a medical degree.
Evidence	Provide a detailed description of student assessment throughout the medical program, including how knowledge, skills, behaviors and attitudes are evaluated. Describe how feedback is part of the assessment system.

2.10 Program evaluation	
Sufficient	There are institutional mechanisms in for evaluating faculty activities, learning settings, teaching methods, and student performance. The results of these evaluations are used to improve the teaching-learning process.
Evidence	Describe the methods and tools used to evaluate teacher activities, disciplines, modules, years of the program and clerkships.

2.11 Integration of curriculum elements	
Sufficient	There is vertical and horizontal integration of curriculum elements and this integration includes theoretical and practical aspects of medicine, different areas of knowledge, diverse levels of heath care and includes psychological, social, cultural, economic and environmental determinants of health.
Evidences	Describe how curriculum integration is designed and performed.

2.12 Clerkships (undergraduate internship)	
Sufficient	Internship takes place at least during two years of medical course in different levels of healthcare system, with clerkships in the following areas: internal medicine, surgery, pediatrics, gynecology-obstetrics, family and community medicine, public health, mental health and emergency medicine, under the supervision of faculty members and/or preceptors with institutional appointment.
Evidence	Provide a detailed description of the clerkships (undergraduate internship) and a critical analysis, including the resources and supervision of medical students and the opportunities of improvement of the quality of clerkships.

**2.13 Elective activities**	
Sufficient	There are elective activities in the curriculum (disciplines, courses and/or clerkships) to complement the core curriculum during medical course and to promote student autonomy in his/her learning process. These elective activities are compatible with curriculum goals and learning outcomes of medical program.
Evidence	Describe elective activities, how they complement the core curriculum and their distribution in the different years of medical program.

**2.14 Complementary activities**	
Sufficient	Complementary activities are regulated and consider the following aspects: workload, diversity of activities and student assessment.<br>*According to Brazilian legislation, complementary activities are learning activities not directly connected to medical curriculum such as language courses, computer training, medical meetings and community projects.
Evidence	Attach the institutional regulations required for complementary activities.

**2.15 Medical research activities**	
Sufficient	Medical school provides to all medical students learning opportunities with the scientific method, including research projects, systematic reviews and/or undergraduate thesis. These activities are regulated concerning learning objectives, hours of activity, supervision and publication of results.
Evidence	Describe the research opportunities offered to medical students during medical course.

2.16 University extension activities	
Sufficient	Medical school provides all students opportunities to participate in university extension activities, regulated and under the supervision of an academic extension committee.<br><br>*According to Brazilian laws, university extension activities are projects and/or programs developed by the university to contribute to the development of local community.
Evidence	Describe the activities of university extension offered to medical students and faculty of medical course. Provide photos, videos, and/or publications.

2.17 Weekly schedule of learning activities	
Sufficient	Weekly distribution of academic activities includes at least two free periods of the day (mornings or afternoons), of four hours, so students can study and/or participate in research, complementary or extension activities, offered by the educational program.
Evidence	For this sub-area, the weekly schedule for each academic year provided in Section II will be evaluated.

2.18 English proficiency	
Sufficient	The educational program provides activities for the use of English language.
Evidence	Describe activities using English language throughout the program.

2.19 Health education	
Sufficient	The educational program provides to medical students learning experiences designed to help individuals and communities to improve their health and/or to support the training of other health professionals.
Evidence	Describe the activities of health education in the curriculum. Provide photos, videos, and/or publications.

2.20 Cultural programs	
Sufficient	Cultural programs that promote various cultural expressions have been available frequently over the past three years.
Evidence	Describe the cultural agenda and activities and the involvement of faculty and medical students. Include photos, videos, and/or publications.

2.21 Academic portal	
Sufficient	Medical school has an electronic communication portal that enables the socialization of institutional information, availability and sharing of educational material, and virtual communication resources.
Evidence	Provide the academic portal address and include a summary of its content and its functionality, describing how information is provided to faculty and medical students. Describe the areas of the curriculum that utilize exclusively virtual learning environment.

2.22 Patient safety	
Sufficient	Patient safety is a fundamental principle of the educational program and promotion of the culture on patient safety can be demonstrated in clinical and learning settings.
Evidence	Describe how patient safety is present in medical curriculum, in disciplines, simulation activities and clinical settings.

Standard 3: Academic Staff/Faculty

Medical school academic staff/faculty is qualified, have experience in the teaching and learning methods, is committed to the production and dissemination of knowledge and participate in actions of faculty development. Medical school values the performance of the preceptors, takes responsibility over their training and provides mechanisms for the inclusion of preceptors in faculty meetings.

**3.1 Faculty qualification (medical school curriculum organized with disciplines or curricular units)**	
Not applicable	
Sufficient	The ratio between faculty members who hold master degree per discipline or curricular unit is equal or greater than 2.0, and doctorate degree is equal or greater than 1.0.
Evidence	Information on the spreadsheet Profile of the Faculty attached to Section II will be analyzed regarding this element.

**3.2 Faculty qualification (medical school curriculum organized with modules)**	
Not applicable	
Sufficient	The ratio between faculty members who hold master degree and module is equal or greater than 4.0, and doctoral degree is equal or greater than 3.0.
Evidence	Information on the spreadsheet Profile of the Faculty attached to Section II will be analyzed regarding this element.

3.3 Educational expertise	
Sufficient	The percentage of teachers of medical program that have expertise in medical education and/or health professions education is equal or greater than 10%.
Evidence	Information on the spreadsheet Profile of the Faculty attached to Section II will be analyzed regarding this element.

3.4 Faculty workload	
Sufficient	The rate of part-time and full-time faculty members is equal or greater than 50% of total faculty members involved in medical course.
Evidence	Information on the spreadsheet Profile of the Faculty attached to Section II will be analyzed regarding this element.

3.5 Faculty appointment and promotion policy	
Sufficient	Medical school has a faculty appointment and promotion policies and they are well known by faculty members. The medical appointment and promotion policies address a balance of capacity of teaching, research and service functions.
Evidence	Describe the policies of faculty appointment and promotion of medical school and provide the institutional norms and/or guidelines. Describe how these policies address a balance of capacity of teaching, research and service functions.

3.6 Student-teacher ratio in practical activities without patients	
Sufficient	The ratio between students and teachers in practical activities that do not include patients is equal or less than 15.
Evidence	Information on the spreadsheet of Educational Planning attached to Section II will be analyzed regarding this element.

3.7 Student-teacher ratio in practical activities with patients	
Sufficient	The ratio between students and teachers in practical activities that include patients is equal or less than six.
Evidence	Information on the spreadsheet of Educational Planning attached to Section II will be analyzed regarding this element.

3.8 Scientific production	
Sufficient	The ratio between the number of book chapters and scientific articles published by the faculty in indexed journals over the past three years and the number of annual medical school vacancies is equal or greater than one.
Evidence	Information on the spreadsheet of Academic Planning attached to Section II will be analyzed regarding this element.

**3.9 Preceptorship**	
Sufficient	Medical school values the performance of the preceptors, takes responsibility over their training and provides mechanisms for the inclusion of preceptors in faculty meetings. The preceptors are familiar with the learning objectives of the course or clerkships and are prepared for their roles in teaching and assessment.
Evidence	Provide a critical analysis about the relationship between the preceptors and medical school and the policies of training and recognition of their role as teachers. The spreadsheet Preceptor Profile attached in Section II will be used to analyze this element.

Standard 4: Students

Medical school promotes a healthy educational environment, positive for learning and personal development. Medical school promotes a culture of institutional resilience, with values of gratitude, generosity, respect and honesty. It provides students with conditions of permanence, health promotion and prevention, access to health services, psychological and pedagogical support. Medical school has clear policies of admission, transfer and student mobility. Students have representation and participation in governance, design and evaluation of the educational program. Medical school approves the presence of representative student organizations and provides proper physical space for them.

**4.1 Selection and admission process**	
Sufficient	Selection process is fair and transparent and considers not only knowledge but also the general competencies of the candidates. The candidates' final scores are subordinated to an institutional policy of inclusion of students underprivileged and disabled.
Evidence	Describe the selective process and social inclusion policies, and attach the public notice of the last selective process.

**4.2 Welcome program**	
Sufficient	Medical school has regulations for welcome receptions of new medical students including follow-up, trial, and punishment for violent behavior. Medical school has a committee that includes faculty and students to organize the reception. It has also institutional campaigns and incentives that promote supportive, non-violent welcome of students. The medical school must demonstrate that no act of embarrassment or violence against new students has taken place over the last three years.
Evidence	Describe the student welcome program. Include photos of the welcome reception to new medical students.

4.3 Programs to support student permanence	
Sufficient	Medical school have programs of scholarships, housing, meals and transportation specific to help underprivileged students.
Evidence	Describe the programs that support student permanence and their range within medical course. Include photos, videos, and/or publications.

4.4 Scholarships	
Sufficient	Medical school offers scholarships for intellectual and scientific development, and social responsibility, with clear mechanisms for admission, permanence and performance assessment. Scholarships include tuition discounts, scientific research, teaching assistance and extension projects.
Evidence	Describe the distribution of scholarships in medical course. Include the last public notices of selections for different types of scholarships.

4.5 Student transference	
Sufficient	Student transference is performed through a selective process that is fair and transparent, considers the general competencies of the candidate and clearly disclosed by public notice.
Evidence	Describe the last process of student transference and provide the public notice.

4.6 Student mobility	
Sufficient	Medical school has student mobility and exchange programs based on formal agreements with at least one national and one international institution.
Evidence	Include the name of each institution that has formal agreements and the number of exchange students enrolled in the previous year, describing origin and destination of them.

4.7 Institutional policies of non-discrimination	
Sufficient	Medical school has institutional policies against any discrimination considering gender, sexual orientation, ethnicity, religious beliefs, age, citizenship and socioeconomic status and there are effective programs and actions.
Evidence	Describe institutional policies and actions that promote non-discrimination considering gender, sexual orientation, ethnicity, religious beliefs, citizenship, age, and socioeconomic status. Include photos, videos, and/or publications.

4.8 Right of student inquiry	
Sufficient	Students have the right for further investigation or appeal on administrative and academic issues, always with a right to defense.
Evidence	Attach the chapter of the statute with the right of students regarding inquiry, recourses and defense.

4. 9 Representativeness	
Sufficient	The council of medical school has student representation and this representation is elected by their peers.
Evidence	Describe how this representation of students occurs in the council of medical School or equivalent instance.

4.10 Student organizations	
Sufficient	Medical school approves the presence of representative student organizations and provides proper physical space for them.
Evidence	Describe the existing representative student organizations and how medical school provides proper physical space for them. Include photos, videos, and/or publications.

4.11 Participation in meetings and congresses	
Sufficient	Medical school support the participation of students in medical education and scientific research congresses and other events.
Evidence	Describe the institutional support to the participation of students in congresses, medical training events, and scientific research events within the last three years. Include photos, videos, and/or publications.

4.12 Students' preventive healthcare	
Sufficient	Immunization reaches every student and medical school has health promotion programs, biosafety protocols and training.
Evidence	Describe preventive healthcare actions implemented for students. Include photos, videos, and/or publications.

4.13 Students' healthcare	
Sufficient	Medical school has an institutional healthcare policy that helps the access of students to medical and dental services for diagnosis, treatment, and preventive healthcare.
Evidence	Describe the offer and access of students to healthcare services.

4.14 Students mental healthcare	
Sufficient	Medical school recognize risk factors for mental illness inherent to medical training and has policies and programs for the psychological support of students, including mental health promotion and mental health assistance services.
Evidence	Describe programs and actions devoted to mental health and psychological support of medical students. Include photos, videos, and/or publications.

4.15 Psycho-pedagogical support	
Sufficient	Medical school provides psycho-pedagogical support to medical students, including mechanisms able to monitor the development and diagnosis of learning difficulties, fulfilling the institutional demand.
Evidence	Describe the psycho-pedagogical support provided to medical students.

4.16 Mentoring programs	
Sufficient	Medical school offers systematic orientation regarding professionalism, human development and citizenship issues, performed by experienced faculty members, with individual and/or group meetings with a maximal number of 20 students per group.
Evidence	Describe the objectives and characteristics of mentoring program(s).

4.17 Quality of life programs	
Sufficient	Medical school has programs designed to promote quality of life, offered to medical students during the entire medical course.
Evidence	Describe the programs regarding quality of life of the students. Include photos, videos and/or publications.

Standard 5: Educational Resources

Medical school has infrastructure, equipment, resources and services that meet the demands of the educational program. Clinical training is performed in health units, outpatient clinics, hospitals and emergency units adequate in number and quality to learning, considering the profile of health problems and the number of patients. There is an adequate maintenance process and concern with the ambience and ecological sustainability.

**5.1 Workspace for faculty**	
Sufficient	Full-time faculty have a fully equipped workspace, shared up to four colleagues, with adequate infrastructure, considering dimension, lighting, cleanliness, acoustics, ventilation, accessibility, maintenance and availability of computer equipment.
Evidence	Describe the workspace allocated for full-time faculty and include photos.

**5.2 Workspace for dean and academic services**	
Sufficient	The space allocated to dean and academic services is adequate considering the following characteristics: size, equipment, conservation, number of employees, and service to students and teachers, respecting ergonomic standards.
Evidence	Describe the dean and academic services workspace and include photos.

**5.3 Teacher workspace**	
Sufficient	Teachers have a shared workspace with adequate infrastructure, considering dimension, lighting, cleanliness, acoustics, ventilation, accessibility, maintenance and availability of computer equipment.
Evidence	Describe the workspace for teachers and include photos.

5.4 Classrooms for large groups	
Sufficient	Classrooms are in adequate number considering the curriculum and have adequate size to accommodate all students plus another 10%; and they have adequate support materials, multimedia, internet access, lighting, acoustics, ventilation, accessibility and maintenance, respecting ergonomic standards.
Evidence	Describe classrooms designed for large groups and include photos.

5.5 Classrooms for small groups	
Sufficient	Rooms designed for small groups are in adequate number considering the needs of curriculum and can accommodate 12 to 15 students, with adequate lighting, ventilation, accessibility, maintenance, and acoustics, respecting ergonomic standards.
Evidence	Describe the classrooms designed for small groups and include photos.

5.6 Teaching laboratories	
Sufficient	Medical school has multidisciplinary laboratories, suitable for the study of different basic aspects of life sciences (anatomy, histology, embryology, biochemistry, pharmacology, physiology/biophysics, surgery and pathology), considering the following: physical space, equipment, safety equipment and supplies. The number of students per equipment or table is adequate for the learning objectives.
Evidence	Describe the teaching laboratories, their equipment and their role in the educational program. Include photos.

5.7 Laboratories of clinical skills and simulation	
Sufficient	Medical school has laboratories with equipment and other resources in sufficient amount to development of clinical skills in all phases of medical course.
Evidence	Describe clinical skills and simulation laboratories and their role in the medical program. Include photos.

5.8 Computer laboratories	
Sufficient	Computer laboratories and other informatics resources are in sufficient number, considering number of equipment, accessibility, and adequacy of physical space and efficient velocity to access internet.
Evidence	Describe the computer laboratories and their insertion in the academic program. Include photos.

5.9 Teaching hospital	
Sufficient	Medical school uses a teaching hospital (or hospitals) accredited by Ministry of Education and Ministry of Health managed by the school or not, it is part of the health system, sufficient for teaching in the areas of Internal Medicine, Surgery, Pediatrics and Gynecology and Obstetrics, with adequate supervision of preceptors.
Evidence	Describe the hospital(s), if it belongs or not to the medical school, if it is accredited, if it is shared by medical students of other medical school, the profile of patients, how it is used by medical course and the relationship between preceptors and medical school. Attach formal agreement terms.

5.10 Teaching outpatient clinics	
Sufficient	The number of outpatient clinics in the areas of Internal Medicine, Surgery, Pediatrics, Gynecology, Obstetrics, and Mental Health is sufficient to comply with the educational program, with a maximum ration of medical students to preceptors of 6.0 and with adequate infrastructure to have the presence of medical students. These outpatient clinics provide an appropriate environment for medical education and training.
Evidence	Describe the outpatient clinics, their infrastructure and the ratio medical students-preceptors. Describe how they provide an appropriate environment for medical education and training.

5.11 Health units/Family health centers	
Sufficient	There are Healthcare units and Family health centers in sufficient number, integrated to the health system, with adequate infrastructure for teaching and sufficient family health teams and preceptors considering the learning objectives of the curriculum. These units provide an appropriate environment for medical education and training.
Evidence	Describe the Health care units and Family health centers, their infrastructure, number of family health teams and how they are integrated with medical course. Describe how they provide an appropriate environment for medical education and training.

**5.12 Emergency training**	
Sufficient	There are emergency services managed by the school or not; they have the infrastructure required for proper care and training. The number of preceptors is sufficient for the medical course. These emergency services provide an appropriate environment for medical education and training.
Evidence	Describe emergency services, whether they are managed by the school or not, their infrastructure, how they are integrated to the health system and the activity of preceptors. Describe how they provide an appropriate environment for medical education and training.

**5.13 Library**	
Sufficient	The library has sufficient space for individual and group study and there is access to the basic bibliography of medical course, either printed or virtual, and meet the needs of medical students and faculty. Library has qualified staff to help the access of students and teachers to virtual and printed material.
Evidence	Describe library and the collection of books and journals and how the library supports the students with the library resources. Include photos.

**5.14 Access to scientific journals**	
Sufficient	There is free access to indexed medical journals by medical students and faculty, adequately distributed among the main areas of knowledge of medical course.
Evidence	Describe the available databases and the access to printed and virtual journals by the medical students and faculty.

5.15 Information and communication technologies	
Sufficient	Information and communication technologies are applied to the teaching-learning process to support the curriculum. There are technical staff to support students and faculty in the use of these technologies
Evidence	Describe the application of information and communication technologies in the teaching-learning process of medical course

5.16 Space for students	
Sufficient	There are areas for study, coexistence, sports and leisure activities of students in sufficient number and size.
Evidence	Describe the areas for study, coexistence, sports and leisure for student activities. Include photos.

5.17 Accessibility	
Sufficient	Medical school facilities ensure accessibility for the people with disabilities, and are equipped to academic activities of students with disabilities.
Evidence	Describe the accessibility regarding people with disabilities. Include photos and/or videos.

5.18 Environmental sustainability	
Sufficient	Medical school has environmental sustainability policies, such as waste separation, reduction of drinking water consumption, water reuse, and prioritizes proper ventilation and natural lighting. Medical school is concerned about the improvement of soil permeability (green areas and permeable paving) and has policies to encourage mobility (rides, bicycles and free shuttle buses).
Evidence	Describe institutional policies of environment sustainability. Include publications, photos and/or videos.
